# Craniofacial Health Care in Southeast Asia: The Unfinished Agenda of Universal Health Coverage

**DOI:** 10.1016/j.identj.2026.109639

**Published:** 2026-05-28

**Authors:** Prasad Nalabothu, Anand Marya, Veersathpurush Allareddy

**Affiliations:** Department of Pediatrics Oral Health and Orthodontics, University Center for Dental Medicine UZB, Basel, Switzerland; Department of Oral and Craniomaxillofacial Surgery, University Hospital Basel, Basel, Switzerland; Department of Orthodontics, Faculty of Dentistry, University of Puthisastra, Phnom Penh, Cambodia; Department of Orthodontics, College of Dentistry, University of Illinois, Chicago, Illinois, USA

Southeast Asia (SEA) has made significant progress toward Universal Health Coverage (UHC), with expanded financial protection and increased access to essential surgical services. However, for children with craniofacial differences, access to surgery alone does not equate to access to comprehensive care. Across the region, craniofacial services remain fragmented, unevenly distributed, and predominantly procedure focused. As a result, functional outcomes often depend less on surgical availability than on the strength of coordinated, multidisciplinary systems. Craniofacial healthcare, therefore, represents an important but under-recognised dimension of UHC implementation.

Congenital anomalies continue to contribute significantly to childhood morbidity and mortality in low- and middle-income settings, including parts of SEA.^1^ While some anomalies are associated with early mortality, conditions such as cleft lip and palate, craniosynostosis, and craniofacial microsomia are compatible with long-term survival but require sustained multidisciplinary management. The Lancet Commission on Global Surgery estimated that 5 billion people lack access to safe and affordable surgical care.^2^ Yet for craniofacial differences, surgical access is only 1 component of effective treatment. Optimal outcomes require coordinated input from speech language therapists, orthodontists, audiologists, geneticists, psychologists, and primary care providers over many years.

In several countries in SEA, primary cleft repair rates have improved, sometimes with support from nongovernmental partnerships. However, the expansion of surgical services has not been matched by proportional growth in allied health capacity.^3^^,^^4^ Workforce shortages are particularly evident in speech and orthodontic services. In Indonesia, for example, the density of speech therapists is substantially lower than in high-income countries, and services are concentrated in major urban centres. Similar geographic disparities are reported in other parts of the region. Consequently, children may receive timely primary repair but lack access to postoperative speech therapy, orthodontic preparation, or secondary procedures such as alveolar bone grafting. Delayed recognition of velopharyngeal insufficiency and untreated malocclusion can compromise long-term functional outcomes and social participation.^4^

Structural barriers persist even in countries with relatively well-developed UHC schemes.^5^ Qualitative evidence from Thailand indicates that caregivers of children with craniofacial anomalies experience fragmented referral pathways, multiple interfacility visits, and indirect financial burdens related to travel and accommodation.^6^ Although services may be covered at the point of care, logistical complexity contributes to discontinuity, particularly for services such as speech therapy that require repeated attendance. Similar concerns regarding care coordination and access to allied services have been documented in Malaysia and Thailand for both cleft and noncleft craniofacial conditions.^7^^,^^8^

The need for coordinated, team-based models of craniofacial care is not new. World Health Organization consultations in the early 2000s emphasised the importance of multidisciplinary services and national registries to support surveillance and quality improvement.^9^ Implementation across SEA remains variable. In many settings, monitoring relies on hospital-based data or records maintained by nongovernmental organisations. Without comprehensive registries capturing prevalence, timing of intervention, and functional outcomes, health systems lack the information necessary to plan workforce development, allocate resources, and evaluate performance beyond surgical volume.

Addressing these gaps requires system-level responses consistent with the broader objectives of UHC.

First, early identification should be integrated into existing primary health care platforms. The Expanded Program on Immunization reaches a high proportion of infants in many countries in SEA.^10^ Incorporating structured assessment of feeding, craniofacial growth, and early speech milestones into routine vaccination visits could facilitate timely referral and reduce delays in accessing specialist services.

Second, workforce development strategies should prioritise allied health integration through hub-and-spoke models. Tertiary craniofacial centres cannot meet regional demand in isolation. Provincial facilities should be supported to provide core services—including speech therapy and orthodontic assessment—with referral linkages and telemedicine support from regional multidisciplinary teams.^11^ Such models may improve geographic equity while maintaining specialist oversight.

Third, performance indicators should extend beyond operative counts. Current UHC monitoring frameworks often emphasise service utilisation and financial protection but rarely assess functional outcomes. For craniofacial care, relevant indicators might include age at primary repair, completion of alveolar bone grafting within recommended time frames, documented speech assessment in early childhood, and continuity of orthodontic follow-up. Embedding these indicators within national health information systems would promote accountability for long-term outcomes rather than procedural outcomes alone.

Craniofacial conditions illustrate a broader principle in health systems strengthening; expanding access to individual interventions does not guarantee continuity of care. Surgical repair, while essential, represents 1 stage in a prolonged developmental pathway. Functional speech, effective mastication, hearing, psychosocial integration, and educational participation depend on coordinated services delivered over time.

As Southeast Asian countries continue to advance toward UHC, attention to multidisciplinary continuity will be critical to ensuring that coverage translates into meaningful health gains. Strengthening allied health capacity, improving data systems, and adopting outcome-oriented metrics can help align craniofacial services with the foundational goals of UHC: equity, quality, and financial protection.

Craniofacial health care should therefore be viewed not as a specialised surgical niche but as a tracer condition for evaluating the depth and integration of UHC systems. Ensuring comprehensive, longitudinal care for children with craniofacial differences represents an important step toward realising the full promise of UHC in the region.

## Conflict of interest

None disclosed.
